# Blood Flow Restriction Does Not Impair Single, All‐Out Sprint Cycling Performance

**DOI:** 10.1002/ejsc.70200

**Published:** 2026-05-28

**Authors:** Carlos Dellavechia de Carvalho, Michelle Stein, Siu Nam Li, Mohammed Ihsan, Peter Peeling, Brendan Richard Scott, Marcelo Papoti, Olivier Girard

**Affiliations:** ^1^ Ribeirao Preto Medical School University of Sao Paulo Ribeirao Preto Brazil; ^2^ School of Human Sciences (Exercise and Sport Science) The University of Western Australia Perth Australia; ^3^ Hypoxia, Sport and Health Team, School of Applied Sciences State University of Campinas Limeira Brazil; ^4^ Physical Education Department College of Education United Arab Emirates University Al Ain UAE; ^5^ Physical Activity Sport and Exercise (PHASE) Research Group School of Allied Health (Exercise Science) Murdoch University Perth Australia; ^6^ Centre for Healthy Ageing Murdoch University Perth Australia; ^7^ School of Physical Education and Sports of Ribeirao Preto University of Sao Paulo Ribeirao Preto Brazil

**Keywords:** cardiorespiratory demands, limb compression, mechanical output, near‐infrared spectroscopy, sprinting

## Abstract

This study examined the effects of sprint duration and blood flow restriction due to cuff pressure applied only during exercise on sprint cycling performance and psychophysiological responses. On separate days, 12 physically active young adults (9 men and 3 women) performed six ‘all‐out’ cycling sprints (5, 10, 15, 20, 25 and 30 s) in a randomised order under three cuff pressure conditions (0%, 40% and 60% of arterial occlusion pressure [AOP]). BFR was applied exclusively during exercise bouts, with passive rest periods of 5–15 min between sprints depending on sprint duration. Power output, peripheral oxygen saturation, *vastus lateralis* muscle oxygenation, heart rate and ratings of perceived exertion were measured. Mean and peak power output and total work done decreased with longer sprints (i.e., 20–30 s) than shorter efforts (i.e., 5–15 s), with no differences observed between cuff pressure conditions. Ventilatory responses, heart rate and muscle oxygenation variables increased with longer sprints. Peripheral oxygen saturation remained unaffected. BFR at 40% and 60% AOP increased limb discomfort for 15‐, 25‐ and 30‐s sprints compared to 0% AOP. In conclusion, increasing the sprint duration decreased mechanical output (external load) and increased physiological strain (internal load), with these effects largely unaffected by moderate‐to‐high (40%–60% AOP) external limb compression.

## Highlights

- A single blood flow–restricted maximal cycling sprint (5–30 s) elicited mechanical outputs comparable to unrestricted exercise bouts.
- Heart rate, oxygen uptake and near‐infrared spectroscopy‐derived muscle oxygenation remained unaffected by external limb compression during sprinting.
- Limb discomfort increased only during longer (≥ 15 s) blood flow‐restricted ‘all‐out’ sprints.

## 1 Introduction

Blood flow restriction (BFR) combined with exercise is a popular training method that involves inflating pneumatic cuffs around the limbs to partially restrict arterial blood inflow and largely occlude venous return (Mckee et al. 2023a; Mouser et al. 2017). This results in augmented muscle deoxygenation and local hypoxia (Ienaga et al. 2022), leading to an accumulation of metabolites such as hydrogen ions and inorganic phosphate (Ienaga et al. 2022; Taylor et al. 2016). Initially used to promote muscle hypertrophy under low mechanical loads in resistance training (Lixandrão et al. 2018) and in rehabilitation settings (Hughes et al. 2017), BFR has more recently been applied to high‐intensity interval training (HIIT) (Keramidas et al. 2012), repeated sprint training (RST) (Giovanna et al. 2022) and sprint interval training (Taylor et al. 2016). However, its acute and chronic effects within these training modalities remain poorly understood.

Recent studies have explored the acute effects of BFR during high‐intensity exercise (Ienaga et al. 2022; Kojima et al. 2021; McClean et al. 2023; Mckee et al. 2024). In particular, continuous BFR applied during RST has consistently been shown to reduce the number of sprints performed to exhaustion and decrease total work or power output (Mckee et al. 2024; Willis et al. 2019; Willis et al. 2018; Willis et al. 2019). These performance decrements are typically accompanied by marked increases in muscle deoxygenation, heart rate and exercise‐related sensations such as limb discomfort and perceived exertion (Li et al. 2023; Smith et al. 2023, 2024). Importantly, emerging evidence suggests that these impairments are not solely explained by peripheral metabolic perturbations; they also reflect altered perceptual responses to exercise. For instance, Smith et al. (2023) demonstrated that trained cyclists maintained a more consistent pace when pedalling under BFR, whereas without restriction, they gradually reduced their pace over time, accompanied by lower rating of perceived exertion (RPE) and cardiovascular demands but greater muscular discomfort and pain. Such interactions between physiological stress and perceptual responses are particularly relevant during all‐out sprint exercise, where tolerance to discomfort and effort perception can strongly influence task completion.

To mitigate excessive strain while preserving the intended physiological stimulus, alternative BFR application strategies have been studied, including BFR applied only during exercise bouts (Giovanna et al. 2022; Mckee, Girard, Peiffer, and Scott 2023a) or exclusively during recovery intervals (Ienaga et al. 2022; Kojima et al. 2021; Taylor et al. 2016). For instance, Kojima et al. (2021) showed that applying BFR during recovery periods in sprint interval training increases *vastus lateralis* muscle deoxygenation without affecting mechanical output. Likewise, Solsona et al. (2022) reported that BFR applied during the first two minutes of recovery at 60% of arterial occlusion pressure (AOP) during a protocol of five ‘all‐out’ 30‐s efforts did not affect peak power output (PPO) or mean power output (MPO), despite a reduced muscle deoxygenation rate (i.e., a slower decline in the tissue saturation index [TSI] at sprint onset) than unrestricted sprints. Finally, Mckee et al. (2023a) demonstrated that continuous BFR impaired performance and increased RPE and limb discomfort, while BFR applied exclusively during very short (≤ 5 s) sprint efforts did not. These findings suggest that performance impairments with BFR application are context‐dependent and more likely to emerge when physiological stress exceeds perceptual tolerance, with the relative contributions of continuous versus intermittent BFR and exercise duration to this stress–performance relationship remaining unclear.

In studies combining BFR with RST, occlusion pressure is typically prescribed using either absolute pressures (i.e., 70–120 mmHg [Ienaga et al. 2022]) or relative pressures expressed as a percentage of individual AOP (i.e., 40%–45% of posterior tibial AOP measured at rest [Mckee et al. 2023b; Patterson et al. 2019]). Relative approaches better account for interindividual variability but require accurate AOP assessment, which may be influenced by cuff characteristics and measurement techniques (Spitz et al. 2024; Yamada et al. 2023). Previous work suggests that 45% AOP may represent the upper tolerable level during repeated short‐duration (i.e., 5–10 s) cycling bouts, based on limb discomfort (Giovanna et al. 2022; Solsona et al. 2024). However, Solsona et al. (2022) reported that higher pressures (up to 60% AOP) may be tolerable when BFR is applied only during recovery between longer (30‐s sprint) efforts.

Recent evidence also supports a dose–response relationship between occlusion pressure and psychophysiological strain, with progressively greater impairments observed as pressure increases. Li et al. (2023) found that ≥ 45% AOP was required to reduce mechanical output during heart rate clamped cycling at the first ventilatory threshold, accompanied by increases in near‐infrared spectroscopy (NIRS)–derived muscle deoxygenation and total hemoglobin (i.e., a surrogate for local blood volume). Notably, at 75% vs. 45%–60% AOP, muscle deoxygenation and total hemoglobin increased further without additional reductions in power output but with disproportionately higher perceptual strain (Li et al. 2023). Although similar ‘dose‐response’ effects have been documented for sprint duration and environmental stressors such as systemic normobaric hypoxia (Weyand et al. 1999), the interactive effects of sprint duration and external limb compression on maximal sprint performance and accompanying exercise responses remain largely unexplored. Hoover et al. (2023) reported decreased power output during a 30‐s Wingate anaerobic test with BFR at 40% AOP, yet it remains unclear whether shorter sprints elicit comparable impairments or whether psychophysiological responses scale proportionally with task duration under BFR.

Therefore, we aimed to determine the acute effects of exercise duration and external limb compression on sprint cycling performance and psychophysiological responses with BFR. We hypothesised that longer sprints would reduce mechanical output and exacerbate psychophysiological responses, particularly at higher occlusion pressures.

## 2 Methods

### 2.1 Participants

Twelve physically active young adults (9 men and 3 women) volunteered for the study (age = 30.3 ± 6.4 years, height = 173.3 ± 10.0 cm, body mass = 75.5 ± 13.4 kg, systolic blood pressure = 113.4 ± 7.1 mmHg and diastolic blood pressure = 70.0 ± 5.5 mmHg). Participants reported exercising 7 ± 1 sessions per week, totalling 555.9 ± 21.0 min across cycling, running, volleyball, tennis, handball and resistance training. The sample size (*n* = 12) was determined a priori using G*Power (Version 3.1.9.7), based on previously reported effect sizes (Willis et al. 2018) of external limb compression during cycling (45% and 60% AOP; effect size = 1.069, power = 0.80 and alpha = 0.05). This sample allows detection of a minimum effect size of *d* ≥ 0.89. Participants were informed of the study requirements and aims, completed the Exercise & Sports Science Australia pre‐exercise screening and provided signed consent. The study received approval from the Human Research Ethics Committee (2023/ET000791) of the host institution.

### 2.2 Experimental Design

This study employed a single‐blind (for occlusion pressure only), within‐participant, crossover design. Participants completed one familiarisation session and three experimental trials, each separated by at least 3 days.

Each session was conducted individually in a controlled environment (22°C) at the same time of day. Female participants (*n* = 3) were tested during the follicular phase (3–10 days after the menstruation onset) of their self‐reported menstrual cycle (Small et al. 2007). During each one of the three experimental sessions, participants performed cycling sprints of varying durations (5, 10, 15, 20, 25 and 30 s) in a randomised order, under three inflatable cuff pressure conditions (0%, 40% and 60% AOP in a randomised order for each effort). Each participant completed 18 randomly assigned conditions (random number generator). During each visit, participants performed one trial at each of the six sprint durations, with each cuff pressure condition applied twice per session. Participants were asked to avoid strenuous exercise, caffeine and alcohol 24 h before testing and to arrive rested and hydrated. During the main experimental trials, participants wore a face mask connected to a metabolic cart for gas exchange analysis, a finger‐mounted pulse oximeter and a wireless NIRS probe positioned over the right *vastus lateralis* muscle. Primary measures included MPO, PPO and work done; secondary measures included oxygen uptake ($\dot{V}O_{2}$), HR, peripheral oxygen saturation, muscle oxygenation and exercise‐related sensations.

### 2.3 Familiarisation Session

Upon arrival at the laboratory, participants' blood pressure was manually measured (ALP K2, Tokyo, Japan) while they laid supine on a padded bench for 10 min. Anthropometric measurements (height, body mass and upper thigh girth) were then taken using a stadiometer, an electronic scale and a tape measure. Participants then underwent a standardized warm‐up similar to that used in the experimental sessions (described below). Following the warm‐up, they performed three maximal efforts lasting 5 s each (unrestricted cycling), with a 5‐min rest between efforts. Finally, participants were familiarised with BFR cycling by undertaking one 30‐s ‘all‐out’ effort with cuff pressure set at 40% AOP, determined using the equation described below. They also received detailed explanations of the perceptual scales during the familiarization session.

### 2.4 Experimental Sessions

**FIGURE 1:**
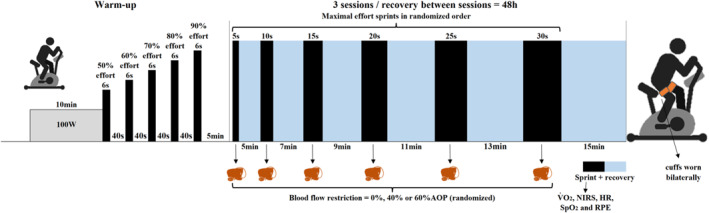
Schematic representation of experimental sessions. AOP = arterial occlusion pressure; HR = heart rate; NIRS = near‐infrared spectroscopy; SpO_2_ = arterial oxygen saturation; $\dot{V}O_{2}$ = oxygen uptake.

Upon arrival, the NIRS participants' measurement site was cleaned and, if necessary, shaved. In all sessions, the device was positioned at the same previously marked location. They then completed a standardised warm‐up, which involved 10 min of continuous cycling at 100 W (RPE < 4 on the 0–10 scale), followed by 1 min of passive rest and followed by five progressive 6‐s submaximal cycling sprints, targeting perceived ‘sense of effort’ levels of 5, 6, 7, 8 and 9 on the modified Bourg CR‐10 scales (Christian et al. 2014). Each bout was interspersed with 40 s of passive recovery (Figure 1). Afterwards, participants rested passively for 5 min while being instrumented with the cuffs, pulse oximeter and face mask.

The sprint cycling protocol consisted of six maximal sprints lasting 5, 10, 15, 20, 25 and 30 s. Each sprint was preceded by a 3‐s countdown and commenced from a stationary start, and participants received strong verbal encouragement throughout. Cuff pressure was applied to both legs immediately before each sprint, inflated to one of three pressure conditions (0%, 40% and 60% AOP) in a randomised order (Figure 1). The sequence of efforts (duration and cuff pressure) for each participant in the three experimental sessions is presented in the supplementary material.

After each exercise bout, participants rested quietly on the cycle ergometer with cuff pressure released. Recovery durations were ≥ 5, 7, 9, 11, 13 and 15 min following 5‐, 10‐, 15‐, 20‐, 25‐ and 30‐s sprints, respectively. In previous testing, these recovery durations were sufficient to restore HR and $\dot{V}O_{2}$ to near pre‐sprint levels. Additionally, rewarming is not required for transitions of ≤ 15 min (Silva et al. 2018). A perceived recovery status scale (Gomes Costa et al. 2023) was administered 30 s before the end of each recovery period. Participants needed to report a score of 8 or above on the 0–10 scale (i.e., indicating ‘well recovered’) before starting the next sprint; otherwise, an additional 60‐s recovery period was provided until the criterion was achieved. This occurred only once, in two participants, after a 30‐s sprint under different conditions.

### 2.5 BFR Application

The BFR was applied bilaterally to the lower limbs using 5‐cm‐wide inflatable cuffs connected to an automated inflation system (Hokanson E20, Bellevue, WA). To ensure blinding and prevent participants from noticing pressure adjustments, the rapid cuff inflator was concealed from view. Cuffs remained on participants even when not inflated and were worn during sprints at 0% AOP. In all cases, cuffs were inflated immediately before each sprint and deflated immediately after with inflation/deflation completed in under 0.3 s.

Thigh circumference and automated brachial blood pressure (HEM‐7203, Omron, Australia) were used to calculate AOP using the following equation: AOP = (5.893 × thigh circumference) + (0.734 × diastolic blood pressure) + (0.912 × systolic blood pressure) − 220.046 (Loenneke et al. 2015).

### 2.6 Responses to Exercise

#### 2.6.1 Mechanical Performance

A custom‐made front‐access cycle ergometer (School of Sport Science, Exercise and Health; University of Western Australia) was calibrated (NATA Ergometer Calibration Rig, Biomed Electronic Services, Australia) and used for all cycling tests. Seat height and handlebar positions were adjusted individually for each participant during familiarisation based on comfort during recovery periods, and these settings were replicated for all experimental trials. MPO, PPO and total work were recorded for each cycling sprint repetition at 1 Hz using customized computer software (Cyclemax, The University of Western Australia, Australia)., and values were expressed relative to body mass.

#### 2.6.2 Physiological Responses

Measurements of $\dot{V}O_{2}$ and minute ventilation were continuously measured using a Parvo TrueOne 2400 metabolic cart (Parvo Medics, Salt Lake City, USA). The metabolic cart was calibrated following the manufacturer's recommendations. Mean $\dot{V}O_{2}$ and minute ventilation values were obtained for each sprint during exercise.

HR and arterial oxygen saturation (SpO_2_) were continuously measured during sprints and recovery periods at the fingertip using pulse oximetry at 1 Hz (IVAC Vital Care DOX Model 506 DXNT, Criticare System Inc., USA). The highest HR recorded was defined as peak HR, while mean HR and SpO_2_ values were obtained for each sprint during exercise.

Muscle oxygenation of the right leg *vastus lateralis* (∼14 cm above the proximal border of the patella) was monitored noninvasively using an NIRS system (Portalite, Artinis Medical Systems, the Netherlands). Measurements were taken from immediately before the first sprint until the end of the recovery period. This system was covered with a piece of black cloth, affixed with tape and reinforced with an elastic bandage. The skin was marked with a permanent marker for accurate probe repositioning. The Portalite unit features three emitter diodes positioned at 30, 35 and 40 mm from the detector, emitting infrared light at 760 and 850 nm wavelengths. All analyses were conducted on data gathered from the 35 mm emitter–detector distance, corresponding to an NIRS signal penetration depth of approximately 17.5 mm within the area of investigation. The Portalite uses both the modified Beer–Lambert and spatially resolved spectroscopy methods to determine changes in oxygenated (O_2_Hb) and deoxygenated (HHb) hemoglobin. Muscle oxygenation changes were interpreted using the TSI (%), an absolute measure of O_2_Hb saturation reflecting the dynamic balance between oxygen demand and supply within the muscle microcirculation (Ihsan et al. 2013). Hemodynamic changes were interpreted using total hemoglobin (tHb = O_2_Hb + HHb), representing local blood volume in micromolar units (μM) (Ferrari et al. 2004). NIRS data were sampled at 10 Hz and downsampled to 1 Hz for analysis. TSI amplitude (maximum TSI − minimum TSI during sprint interval) and integrated TSI ([baseline TSI − average TSI during sprint interval] × sprint duration) were calculated to characterise muscle deoxygenation for each sprint duration. Changes in local blood volume during sprints were characterised by tHb amplitude (i.e., maximum tHb during sprint interval − minimum tHb at sprint onset) (Ihsan et al. 2013).

#### 2.6.3 Ratings of Perceived Exertion and Limb Discomfort

Ratings of overall perceived exertion and perceived lower limb discomfort were recorded, using a modified Bourg CR10 scale (Christian et al. 2014), by asking the questions: ‘What is your overall perceived exertion?’ (Participants were instructed to consider overall perceived exertion, including sensations in the legs.) and ‘How heavy do your legs feel?’, respectively. These perceptual measures were collected immediately after each sprint in an invariant order.

### 2.7 Statistical Analysis

Data distribution was assessed via a Shapiro–Wilk test. Exercise‐related sensations presented a nonnormal distribution. For normally distributed data, a two‐way analysis of variance for repeated measures was used to investigate the main effect of sprint duration and the occlusion pressure, as well as their interaction. Nonnormally distributed data were analysed using Friedman's nonparametric test. Effect sizes were reported as partial eta‐squared (*η*_p_^2^, with *η*_p_^2^ ≥ 0.06 representing a *moderate* effect and *η*_p_^2^ ≥ 0.14 a *large* effect). Bonferroni post hoc pairwise comparisons were used to locate significant effects. Statistical testing was carried out using IBM SPSS Statistics 26. The alpha level was set at *p* < 0.05. All data are presented as group mean ± SD.

## 3 Results

### 3.1 Familiarization Session

Power output during 5‐s and 30‐s sprints did not differ between familiarization and experimental sessions (*p* > 0.05). Participants had a thigh circumference of 59.0 ± 5.3 cm, systolic and diastolic pressures of 113.4 ± 7.1 mmHg and 70.0 ± 7.1 mmHg, respectively, and an estimated AOP of 264.8 ± 34.8 mmHg.

### 3.2 Performance Outcomes

**FIGURE 2:**
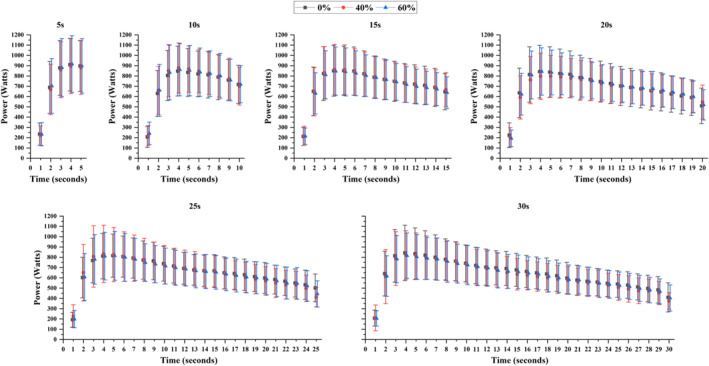
Absolute power for each second through all sprint's durations and for the three occlusion pressure conditions. Data are presented as mean ± standard deviation (*n* = 12).

Significant effects were observed for the sprint duration across all power output variables (*F* > 8.873, *p* < 0.001 and *η*_p_^2^ > 0.443; Table 1, and Figure 2). There were no significant interactions or any main effects of cuff pressure (*F* < 0.726, *p* > 0.496 and *η*_p_^2^ < 0.230; Table 1, and Figure 2). Post hoc analyses showed that PPO was higher during the 5‐s sprint than all longer durations (*p* < 0.035). PPO during the 10‐s sprint was greater than the 20‐ and 25‐s sprints (*p* < 0.043), while PPO during the 15‐s sprint was higher than during the 25‐s effort (*p* = 0.030). MPO during the 5‐s sprint was higher than during the 25‐ and 30‐s efforts (*p* < 0.016). MPO during the 10‐s sprint was higher than during the 20‐, 25‐ and 30‐s efforts (*p* < 0.001). MPO during the 15‐, 20‐ and 25‐s sprints was higher than during the longest effort duration (*p* < 0.037). Absolute work increased with sprint duration (*p* < 0.001).

**TABLE 1:** Mechanical performance responses for six sprint durations and the three blood flow restriction conditions.

	5s	10s	15s	20s	25s	30s	Repeated measures ANOVA		
							Duration	Pressure	Interaction
Peak power (Watts·kg^−1^)									
0%	12.5 ± 2.5^a^	11.6 ± 2.0^b^, ^c^	11.9 ± 2.4^c^	11.7 ± 2.3	11.4 ± 1.6	11.7 ± 2.8	*F* = 8.909	*F* = 0.238	*F* = 0.548
40%	12.2 ± 2.4^a^	12.1 ± 2.4^b^, ^c^	12.0 ± 2.5^c^	11.3 ± 1.9	11.6 ± 2.8	11.5 ± 2.3	**p < 0.001**	*p* = 0.790	*p* = 0.852
60%	12.5 ± 2.5^a^	11.9 ± 2.5^b^, ^c^	11.7 ± 2.4^c^	11.7 ± 2.2	11.5 ± 2.5	11.3 ± 1.9	*η*_p_^2^ = 0.447	*η*_p_^2^ = 0.021	*η*_p_^2^ = 0.047
Mean power (Watts·kg^−1^)									
0%	9.5 ± 2.3^c^, ^d^	9.4 ± 1.7^b^, ^c^, ^d^	9.5 ± 1.8^a^	9.0 ± 1.6^a^	8.6 ± 1.3^a^	8.4 ± 1.5	*F* = 14.904	*F* = 0.300	*F* = 0.488
40%	9.2 ± 2.1^c^, ^d^	9.8 ± 1.9^b^, ^c^, ^d^	9.5 ± 1.7^a^	8.9 ± 1.4^a^	8.5 ± 1.4^a^	8.1 ± 1.4	**p = 0.001**	*p* = 0.744	*p* = 0.894
60%	9.5 ± 2.3^c^, ^d^	9.7 ± 2.1^b^, ^c^, ^d^	9.4 ± 1.8^a^	9.0 ± 1.4^a^	8.5 ± 1.5^a^	8.2 ± 1.4	*η*_p_^2^ = 0.598	*η*_p_^2^ = 0.029	*η*_p_^2^ = 0.047
Work (kilojoules)									
0%	3.6 ± 1.1^a^	7.2 ± 2.0^a^	10.9 ± 2.8^a^	13.7 ± 3.6^a^	16.3 ± 3.8^a^	19.1 ± 4.5	*F* = 184.463	*F* = 0.726	*F* = 0.310
40%	3.5 ± 1.1^a^	7.4 ± 1.9^a^	10.9 ± 2.7^a^	13.4 ± 3.2^a^	16.1 ± 4.0^a^	18.5 ± 4.2	**p < 0.001**	*p* = 0.496	*p* = 0.864
60%	3.6 ± 1.1^a^	7.4 ± 2.0^a^	10.7 ± 2.6^a^	13.7 ± 3.2^a^	16.0 ± 3.8^a^	18.6 ± 4.4	*η*_p_^2^ = 0.949	*η*_p_^2^ = 0.068	*η*_p_^2^ = 0.030

*Note:* Data are presented as mean ± standard deviation (*n* = 12). The bold values​ indicate statistical significance.

Abbreviations: F = statistic value from the ANOVA.

^a^ Different from all longer sprints.

^b^ Different from 20‐s sprints.

^c^ Different from 25‐s sprints.

^d^ Different from 30‐s sprints.

### 3.3 Physiological Responses

**FIGURE 3:**
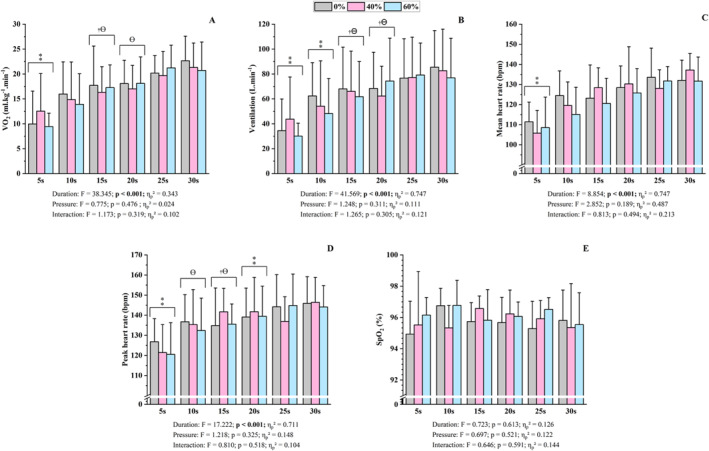
Physiological responses for six sprint durations and for the three blood flow restriction conditions. Data are presented as mean ± standard deviation (*n* = 12). A = $\dot{V}O_{2}$ (oxygen uptake); B = ventilation; C = mean heart rate; D = peak heart rate; E = arterial oxygen saturation; F = statistic value from the ANOVA; ⁑ = different from all longer sprints; † = different from 25‐s sprints; Ɵ = different from 30‐s sprints. Data are presented as mean ± standard deviation (*n* = 12).

A significant effect of sprint duration was observed for $\dot{V}O_{2}$, minute ventilation and both mean and peak HR (*F* = 5.93, *p* < 0.001 and *η*_p_^2^ > 0.458, Figure 3A–D). No significant main effects of cuff pressure or sprint duration × cuff pressure interactions were detected. Post hoc analyses indicated that $\dot{V}O_{2}$, VE, and both peak and mean HR during the 5‐s sprint were lower than during all longer efforts (*p* < 0.046). $\dot{V}O_{2}$ during the 15‐s sprint was lower than during the 25‐ and 30‐s sprints (*p* < 0.028), and $\dot{V}O_{2}$ during the 20‐s was lower than during the 30‐s effort (*p* = 0.001). VE during the 10‐s sprint was lower than during all longer effort durations (*p* < 0.007). Peak HR during the 10‐s sprint was lower than during the 30‐s effort (*p* = 0.039), and both VE and peak HR during the 15‐ and 20‐s sprints were lower than during the 25‐ and 30‐s efforts (*p* < 0.035). SpO_2_ during sprints demonstrated no significant effects (*F* = 0.686, *p* = 0.809 and *η*_p_^2^ > 0.121, Figure 3E).

**FIGURE 4:**
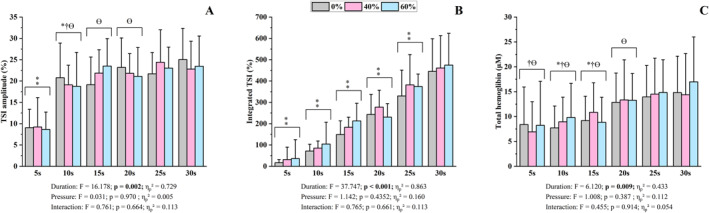
Near‐infrared spectroscopy responses for six sprint durations and the three blood flow restriction conditions. Data are presented as mean ± standard deviation (*n* = 12). TSI = tissue saturation index; A = TSI amplitude; B = integrated TSI; C = total hemoglobin; F = statistic value from the ANOVA; ⁑ = different from all longer sprints; * = different from 20‐s sprints; † = different from 25‐s sprints; Ɵ = different from 30‐s sprints.

For the NIRS‐derived variables, significant effects were observed only for sprint duration in TSI amplitude, integrated TSI and total hemoglobin (*F* = 6.12, *p* < 0.009 and *η*_p_^2^ > 0.433, Figure 4). No main effects of cuff pressure or sprint duration × cuff pressure interactions were noted. TSI amplitude during the 5‐s sprint was lower than during all longer efforts (*p* < 0.013). TSI amplitude during the 10‐s sprint was lower than during the 20‐, 25‐ and 30‐s efforts (*p* < 0.027), while values during the 15‐ and 20‐s sprints were lower than during the 30‐s effort (*p* < 0.050). Integrated TSI increased with sprint duration (*p* < 0.014). Total hemoglobin during the 5‐s sprint was lower than during the 25‐ and 30‐s efforts (*p* < 0.044). Values during the 10‐ and 15‐s sprints were lower than during the 20‐, 25‐ and 30‐s efforts (*p* < 0.017), and values during the 20‐s sprint were lower than during the 30‐s effort (*p* < 0.038).

### 3.4 Exercise‐Related Sensations

**FIGURE 5:**
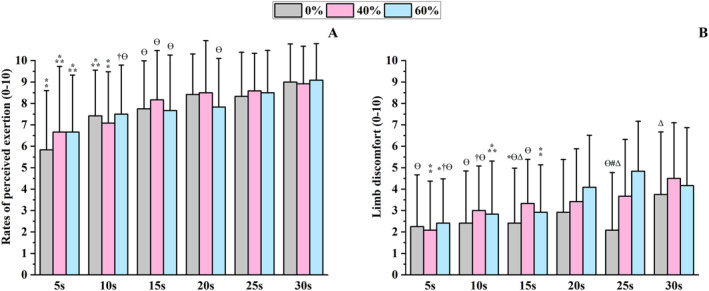
Ratings of perceived exertion (A) and limb discomfort (B) for six sprint durations and three blood flow restriction conditions. Data are presented as mean ± standard deviation (*n* = 12). A = ratings of perceived exertion; B = limb discomfort; ⁑ = different from all longer sprints at the same pressure; ⁂ = different from all longer sprints at the same pressure, except the immediately longer; * = different from 20‐s sprint at the same pressure; † = different from 25‐s sprint at the same pressure; Ɵ = different from 30‐s sprint at the same pressure; Δ = different for 40% sprint in the same duration; # = different for 60% sprint in the same duration.

There was a main effect of the sprint duration for RPE (*χ* ^2^ = 89.7 and *p* < 0.001, Figure 5A). Post hoc analyses showed that RPE during the 5‐ and 10‐s sprints was lower than during most longer effort durations (*p* < 0.012), while RPE during the 15‐ and 20‐s sprints were lower than during the 30‐s effort (*p* < 0.050). Significant effects of the sprint duration and cuff pressure × sprint duration interaction were noted for limb discomfort (*χ* ^2^ = 80.8 and *p* < 0.001, Figure 5B). Limb discomfort during 5‐, 10‐ and 15‐s sprints was lower than during most longer efforts (*p* < 0.014). Additionally, in the 15‐ and 30‐s sprints, discomfort at 0% AOP was lower than at 40% AOP (*p* = 0.040), while for the 25‐s sprint, discomfort at 0% AOP was lower than at both 40% and 60% AOP (*p* < 0.001).

## 4 Discussion

This study investigated the effects of the sprint duration (5–30 s) and BFR cuff pressure (0%–60% AOP) on performance, physiological and perceptual responses. The main findings were as follows: (i) Longer sprints resulted in progressively lower mechanical power and higher physiological responses than shorter sprints. (ii) BFR (40%–60% AOP) did not significantly affect these outcomes. (iii) BFR elevated limb discomfort during 15‐, 25‐ and 30‐s sprints compared to the non‐BFR condition, while other perceptual responses were primarily influenced by sprint duration. These findings partly support our hypothesis, indicating that longer sprints reduced mechanical output (external load) and increased physiological responses (internal load), with minimal effects from low‐to‐moderate BFR cuff pressures.

Power output decreased progressively across sprints as their duration increased, with higher MPO observed in shorter sprints, as expected. This decline is attributed to fatigue from intense muscular effort (Pekünlü et al. 2016). The lack of an effect from cuff pressure here contrasts with Hoover et al. (2023), who reported lower speed and power in a single 30‐s ‘all‐out’ effort with BFR than unrestricted conditions. However, our results align with Mckee et al. (2023a), who reported no impairments in repeated‐sprint performance with BFR during work intervals, although continuous BFR did affect performance in 5‐s efforts. We extend these findings by demonstrating that BFR, even up to 60% AOP, does not impair performance in longer sprints (up to 30 s), despite 40% AOP being commonly prescribed (Smith et al., 2022). Consequently, BFR does not adversely affect performance outcomes in 5–30 s cycling sprints under conditions of near‐complete recovery.

We observed that $\dot{V}O_{2}$, ventilation and HR increased with sprint duration but remained unaffected by BFR. These results align with Mckee et al. (2023a), who showed no significant differences in these variables between BFR (45% AOP) and nonBFR conditions during an RST session. Notably, these physiological variables primarily rely on aerobic metabolism (Poole and Jones 2012), which is not fully activated during short sprints due to the transient nature of ventilatory responses, causing a delay in aerobic activation (Poole and Jones 2012; Stirling et al. 2005). This supports the dominance of anaerobic metabolism during 5–30 s sprints, where adenosine triphosphate (ATP) resynthesis via anaerobic sources (i.e., phosphocreatine and anaerobic glycolysis) occurs more rapidly than through aerobic pathways (Hargreaves and Spriet 2020). Our findings are consistent with studies demonstrating no decrements in power output with intermittent BFR during short sprints such as five 10‐s sprints with 40‐s passive rest (Kojima et al. 2021), three sets of three 6‐s maximal sprints with 24 s of rest (Ienaga et al. 2022) or three sets of five 5‐s maximal sprints with 25 s of rest (Mckee, Girard, Peiffer, and Scott 2023a). In contrast, continuous BFR impairs PPO and MPO during a 30‐s single sprint (Hoover et al. 2023) and during five 30‐s sprints interspaced with 4 min of passive recovery, resulting in lower $\dot{V}O_{2}$, ventilation and HR (Solsona et al. 2021). Similar attenuated systemic physiological responses were noted by McClean et al. (2023) during repeated‐sprint training; however, when BFR was applied intermittently and only during exercise, performance did not differ from non‐BFR conditions. Collectively, the evidence suggests that continuous BFR compromises both systemic physiological variables and performance, likely due to BFR‐mediated restrictions in arterial inflow and venous flow (Early et al. 2020), delaying reoxygenation and aerobic ATP resynthesis. Nevertheless, continuous BFR may also affect the validity of indirect calorimetry measurements, potentially influencing these conclusions (Walden et al. 2022).

In this study, muscle deoxygenation (measured as TSI amplitude and integrated TSI) and local blood volume (tHb amplitude) generally increased with sprint duration, reflecting a greater oxygen extraction (Bogdanis et al. 1998; Gastin 2001). Contrary to our hypothesis, cuff pressure did not affect any NIRS‐derived measures. This differs from recent studies reporting greater muscle deoxygenation and local blood volume with BFR or higher cuff pressures (Behrendt et al. 2023; Ienaga et al. 2022; Li et al. 2023; Willis et al. 2018). However, these studies involved submaximal exercise (i.e., HR‐clamped at the first ventilatory threshold) (Li et al. 2023), multiple sprints (Behrendt et al. 2023; Mckee et al. 2024), sprints to exhaustion (Willis et al. 2018) or BFR applied during recovery intervals (Ienaga et al. 2022). Our study offers novel insights into NIRS‐derived indices to different BFR pressures during single sprints of varying durations. The lack of BFR effects on muscle oxygenation measures suggests that the aerobic demands of 5–30s sprints were not impacted by circulatory limitations imposed by BFR at 40%–60% AOP. As such, aerobic metabolism likely remained secondary, maintaining a stable deoxygenation profile despite external limb compression. Consequently, applying BFR at 40%–60% AOP does not exaggerate muscle oxygen demand during single sprints lasting 5–30s.

Consistent with mechanical load and physiological variables, different occlusion pressures did not affect RPE. BFR during single sprints seems tolerable; however, Mckee et al. (2023a) reported increased perceptual strain with continuous BFR across multiple sprints. However, RPE values were higher following longer sprints, probably due to the linear relationship between RPE and workload (Balagué et al. 2015). Sprint duration also affected limb discomfort, whereas the effects of cuff pressure were more circumstantial. Additionally, the participants were unable to distinguish between the cuff pressures used, so their mental ability to perform the exercise task or future tasks does not seem to be affected. Our results suggest that higher limb discomfort caused by BFR does not affect performance in ‘all‐out’ single sprints up to 30s. For instance, McClean et al. (2023) and Mckee et al. (2023a) showed greater local pain with continuous BFR (60% and 45% AOP, respectively) in HIIT and RST protocols than unrestricted conditions. Conversely, Wang et al. (2023) observed no detrimental effect with continuous BFR (140 mmHg) in RST. With intermittent BFR, Mckee et al. (2023a) showed no difference in limb discomfort compared to unrestricted conditions. These findings suggest that the impact of BFR on perceptual measurements may depend on cuff pressure, application method (i.e., continuous or intermittent) and individual tolerance.

This study has several limitations. First, the sex imbalance (i.e., 9 men and 3 women) may limit the generalisability of the results to women. McClean et al. (2023) reported that BFR affects neuromuscular, perceptual and cardiorespiratory responses more in females than in males. Additionally, the menstrual cycle phase was not directly measured in female participants. Second, participants were informed of sprint durations before each trial. Despite being instructed to exert maximum effort, peak power during 5‐s sprints was higher than in longer ones. This suggests that participants may have subconsciously restrained their maximal power output during longer sprints, likely due to the psychobiological challenge of an ‘all‐out’ effort and the anticipated impact on performance (Blanchfield et al. 2014). Furthermore, applying six efforts within the same experimental session may produce, although not observed, residual effects on the execution of subsequent sprints. This also prevents the implementation of a fully counterbalanced design, which may amplify the effects of confounding factors and order. The selected analysis period (i.e., restricted to the sprint duration) may not fully capture physiological stress, particularly for ventilatory variables, during shorter sprints. Third, subcutaneous adipose tissue thickness at the NIRS site was not assessed, which may affect measurements, particularly for female participants. Finally, AOP was estimated indirectly, potentially reducing accuracy, as recent studies indicate that narrow cuffs (i.e., such as the 5‐cm wide cuffs used here) may not accurately measure AOP (Spitz et al. 2024; Yamada et al. 2023), which also varies with cuff size and body position (de Queiros et al. 2024). Notably, complete arterial occlusion was not achieved in all participants during the equation‐based estimation procedure. Thus, caution is needed when using this approach in individuals with larger limb circumferences, as full occlusion may not be reached during measurement (Loenneke et al. 2015).

## 5 Conclusion

These findings suggest that longer sprints (i.e., 20–30 s) reduce mechanical output and heighten psychophysiological responses than shorter sprints (i.e., 5–15 s), irrespective of BFR cuff pressure. Applying BFR during maximum single sprints does not compromise performance. Thus, using BFR during an isolated maximal sprint (< 30s) enables athletes to sustain performance and key physiological responses, despite increased limb discomfort.

## Funding

This study was possible thanks to the Federal Agency for Support and Evaluation of Graduate Education (Coordenação de Aperfeiçoamento de Pessoal de Nível Superior, CAPES) through the program CAPES‐PrInt (88887.835398/2023‐00). Authors CDC, GMP, and MP are supported by São Paulo Research Foundation (Fundação de Amparo à Pesquisa do Estado de São Paulo, FAPESP) through the grants Thematic project (2023/02728‐3) and Technical training (2026/02573‐8). Author BRS is supported by an Investigator Grant from the National Health and Medical Research Council (APP1196462).

## Conflicts of Interest

The authors declare no conflicts of interest.

## Supporting information


Supporting Information S1

## Data Availability

The data that support the findings of this study are available from the corresponding author upon reasonable request.
