# Supplementary material for: Blood Flow Restriction Does Not Impair Single, All‐Out Sprint Cycling Performance
Source: Eur J Sport Sci. 2026 May 28;26(6):e70200. doi: 10.1002/ejsc.70200 (PMC13239908; doi:10.1002/ejsc.70200)
Supplement: Supplementary file 1 — Supporting Information S1 [file EJSC-26-e70200-s001.pdf]

Supplementary material: Sequence of efforts (duration and cuff pressure) for each participant in the three experimental sessions.

Sessions	P1	P2	P3	P4	P5	P6	P7	P8	P9	P10	P11	P12
1ª	5s40%	10s60%	10s60%	25s60%	5s40%	25s60%	30s60%	15s0%	15s60%	20s40%	30s0%	30s60%
	15s60%	15s60%	25s40%	5s60%	10s0%	10s0%	15s0%	30s60%	20s0%	25s40%	5s0%	10s40%
	30s60%	30s0%	20s0%	20s40%	20s0%	30s60%	20s60%	10s60%	25s60%	5s60%	15s40%	25s0%
	10s0%	5s40%	15s60%	15s0%	30s40%	5s0%	5s40%	20s0%	30s40%	30s0%	25s60%	15s0%
	20s40%	25s40%	30s40%	10s0%	15s60%	15s40%	10s0%	25s40%	10s40%	15s60%	10s40%	20s40%
	25s0%	20s0%	5s0%	30s40%	25s60%	20s40%	25s40%	5s40%	5s0%	10s0%	20s60%	5s60%
2ª	30s40%	20s40%	5s60%	20s0%	20s60%	5s40%	15s60%	30s40%	15s0%	30s60%	20s0%	15s60%
	15s40%	5s60%	30s0%	15s60%	25s40%	20s60%	5s60%	15s60%	10s60%	20s0%	5s60%	5s40%
	20s0%	25s0%	10s40%	30s60%	15s40%	10s40%	10s40%	5s0%	30s60%	15s40%	25s40%	10s0%
	25s60%	30s60%	25s60%	25s40%	10s60%	25s0%	25s0%	25s0%	25s0%	10s40%	10s60%	30s40%
	10s60%	10s40%	20s40%	5s0%	5s0%	15s60%	20s0%	20s60%	5s40%	25s60%	15s0%	20s0%
	5s0%	15s0%	15s0%	10s40%	30s0%	30s0%	30s40%	10s40%	20s40%	5s0%	30s40%	25s60%
3ª	25s40%	20s60%	15s40%	5s40%	25s0%	5s60%	10s60%	15s40%	15s40%	25s0%	25s0%	5s0%
	30s0%	25s60%	20s60%	10s60%	10s40%	25s40%	20s40%	25s60%	5s60%	15s0%	5s40%	20s60%
	5s60%	5s0%	30s60%	25s0%	15s0%	20s0%	5s0%	20s40%	30s0%	20s60%	30s60%	15s40%
	10s40%	30s40%	25s0%	15s40%	5s60%	30s40%	25s60%	30s0%	20s60%	30s40%	10s0%	25s40%
	15s0%	10s0%	10s0%	20s60%	20s40%	10s60%	30s0%	5s60%	25s40%	5s40%	15s60%	10s60%
	20s60%	15s40%	5s40%	30s0%	30s60%	15s0%	15s40%	10s0%	10s0%	10s60%	20s40%	30s0%

P = Participant.
